# Penalties for Emergency Medical Treatment and Labor Act Violations Involving Obstetrical Emergencies

**DOI:** 10.5811/westjem.2019.10.40892

**Published:** 2020-02-21

**Authors:** Sophie Terp, Brandon Wang, Elizabeth Burner, Sanjay Arora, Michael Menchine

**Affiliations:** * Keck School of Medicine, University of Southern California, Los Angeles, California; † New York University School of Medicine, New York, New York

## Abstract

**Introduction:**

The Emergency Medical Treatment and Labor Act (EMTALA) was intended to prevent inadequate, delayed, or denied treatment of emergent conditions by emergency departments (ED). While controversies exist regarding the scope of the law, there is no question that EMTALA applies to active labor, a key tenet of the statute and the only medical condition – labor – specifically included in the title of the law. In light of rising maternal mortality rates in the United States, further exploration into the state of emergency obstetrical (OB) care is warranted. Understanding civil monetary penalty settlements levied by the Office of the Inspector General (OIG) related to EMTALA violations involving labor and other OB emergencies will help to inform the current state of access to and quality of OB emergency care.

**Methods:**

We reviewed descriptions of all EMTALA-related OIG civil monetary penalty settlements from 2002–2018. OB-related cases were identified using keywords in settlement descriptions. We described characteristics of settlements including the nature of the allegation and compared them with non-OB settlements.

**Results:**

Of 232 EMTALA-related OIG settlements during the study period, 39 (17%) involved active labor and other OB emergencies. Between 2002 and 2018 the proportion of settlements involving OB emergencies increased from 17% to 40%. Seven (18%) of these settlements involved a pregnant minor. Most OB cases involved failure to provide screening exam (82%) and/or stabilizing treatment (51%). Failure to arrange appropriate transfer was more common for OB (36%) compared with non-OB settlements (21%) (p = 0.041). Fifteen (38%) involved a provider specifically directing a pregnant woman to proceed to another hospital, typically by private vehicle.

**Conclusion:**

Despite inclusion of the term “labor” in the law’s title, one in six settlements related to EMTALA violations involved OB emergencies. One in five settlements involved a pregnant minor, indicating that providers may benefit from education regarding obligations to evaluate and stabilize minors absent parental consent. Failure to arrange appropriate transfer was more common among OB settlements. Findings suggesting need for providers to understand EMTALA-specific requirements for appropriate transfer and for EDs at hospitals without dedicated OB services to implement policies for evaluation of active labor and protocols for transfer when indicated.

## INTRODUCTION

The Emergency Medical Treatment and Labor Act (EMTALA) was enacted in 1986, in response to highly publicized incidents of inadequate, delayed, or denied treatment of uninsured patients including pregnant women by emergency departments (ED).^1^–^4^ While controversies exist regarding the scope of EMTALA,^5^ there is no question that the law applies to active labor, a key tenet of the statute and the only condition specifically included in the title of the law. EMTALA is actively enforced with more than a quarter of hospitals in the United States having received citations for EMTALA violations between 2005–2014.^6^

More than three decades after passage of EMTALA, hospitals continue to be cited for EMTALA cases related to labor and other obstetrical (OB) emergencies. Between 2005–2014, 198 (9%) of 2118 citations for EMTALA violations were related to labor and 97 (5%) to other OB emergencies.^6^ Prior systematic studies have described general patterns of EMTALA enforcement,^6^ resulting fines,^7^–^10^ impact of the law on on-call coverage,^11^ and patterns of EMTALA transfers for surgical subspecialty care.^12^–^17^ Despite the fact that labor is the only medical condition named in the title of the law, EMTALA violations related to labor and other OB emergencies have not previously been systematically described in the peer-reviewed medical literature.

EMTALA requires that all patients presenting to a dedicated ED have 1) a timely medical screening, 2) stabilization of emergency medical conditions, and 3) transfer of care if services required for stabilization are not available at the facility.^18^ Hospitals have a duty to accept transfer of patients requiring specialty care if the facility has an on-call specialist and capacity to treat the patient.^18^ All hospitals with Medicare provider agreements are subject to EMTALA, and enforcement is conducted by the Centers for Medicare and Medicaid Services (CMS). The Office of the Inspector General (OIG) of the Department of Health and Human Services has power to assign civil monetary penalties to facilities and individual physicians that violate EMTALA.^19^ An estimated 7.9% of EMTALA violations result in a civil monetary penalty.^9^ The historic maximum civil monetary penalty of $50,000^18^ for an EMTALA violation increased to $103,139 in 2016.^20^

While general characteristics of OIG civil monetary penalties have been previously described for hospitals^7^,^9^,^10^ and individual physicians,^8^ characteristics of civil monetary penalties related to EMTALA violations involving active labor and other OB emergencies specifically have not previously been described. In light of rising maternal mortality rates in the US^21^ that now far exceed those of other developed countries, further exploration into the state of emergency OB care is warranted. Understanding civil monetary penalty settlements levied by the OIG related to EMTALA violations involving labor and other OB emergencies will help to inform the current state of access to and quality of emergency care for patients with labor and other OB emergencies. The purpose of this study is to describe characteristics of civil monetary penalties imposed by the OIG related to EMTALA violations involving active labor and other OB emergencies.

**Population Health Research Capsule**

What do we already know about this issue?

While labor is the only condition named in the law’s title, EMTALA violations related to labor and other obstetrical (OB) emergencies have not previously been described.

What was the research question?

To describe characteristics of civil monetary penalties related to EMTALA violations involving labor and other OB emergencies.

What was the major finding of the study?

One in six settlements involved OB cases (one in five were pregnant minors). OB settlements more often involved failure to arrange transfer.

How does this improve population health?

Providers may benefit from education regarding EMTALA requirements to evaluate, stabilize and, when necessary, arrange appropriate transfer of patients with OB emergencies.

## METHODS

### Study Design and Data Sources

We obtained case descriptions of all civil monetary penalty settlements issued between 2002–2018 from the OIG.^22^ Using methodology established in prior work,^7^,^8^ we identified civil monetary penalty settlements related to EMTALA violations by inclusion of the terms “EMTALA” or “patient dumping” in the title or text of the settlement description, and settlements unrelated to EMTALA (eg, kickback allegations, fraudulent Medicare claims) were excluded from analysis. Case descriptions included settlement amount, location, and brief description of the involved patient’s condition and for some cases, clinical course, although the level of detail provided varied between entries. We additionally categorized locations by CMS region, the level at which EMTALA is enforced. Appendix A includes a map depicting each of the 10 CMS regions.

### Identification of Cases Involving Obstetrical Emergencies

We identified settlements related to OB conditions by searching text of case settlement descriptions for key words: pregnant, pregnancy, birth, and labor. We excluded cases where the term “labor” was included in the description as part of the EMTALA acronym without relevance to an OB context. Each case description was reviewed and coded by two authors (EB, ST), and kappa statistics were calculated to evaluate for inter-rater reliability for identification of OB cases.^23^

### Recording of Case Features

We recorded the date, location, and settlement amount for each case, as well as whether the settlement involved a hospital or individual physician. When available, the age of involved patient and location of the incident within the hospital were recorded as well (ED vs labor and delivery triage). Settlement descriptions were reviewed to determine if they described 1) failure to provide appropriate medical screening exam, 2) failure to provide stabilizing treatment, 3) failure to arrange appropriate transfer, 4) failure to accept appropriate transfer, or 5) failure of an on-call doctor to respond, consistent with prior work in this field.^7^ These categories correspond to EMTALA deficiency tags involving clinical aspects of care, and a list of tags and descriptions is included in Appendix B.

Of note, for settlement descriptions describing EMTALA deficiencies for both an OB patient and a non-OB patient, only those deficiencies involving the OB patient were included in analysis. For example, in one case a Florida hospital system agreed to pay $85,000 for allegedly violating the Patient Anti-Dumping Statute on three separate occasions when they did the following: 1) inappropriately transferred a 27-year old female in active labor; 2) did not accept a patient referred to one of its facilities under the Baker Act; and 3) failed to provide an appropriate medical screening examination for a patient who arrived at its ED. For the present analysis, only the first instance, the inappropriate transfer of the patient in active labor, would have been recorded as the failure to accept and failure to provide a medical screening exam pertained to non-OB patients. Settlement descriptions involving labor and OB emergencies were systematically reviewed for 1) reference to a provider directing a pregnant patient to proceed to another facility, 2) whether they were directed to the facility where their obstetrician practiced, and 3) whether the transport was by private vehicle.

### Data Analysis

We compared characteristics of cases resulting in OIG settlements between those involving and those not involving OB emergencies with t-tests, chi-squared and Fisher’s exact tests, as indicated. We performed statistical analyses using Stata/MP13 (StataCorp, College Station, TX). This study was approved by the Health Sciences Institutional Review Board at the University of Southern California, Los Angeles.

### Illustrative Case Study

To provide a richer understanding of EMTALA violations, enforcement and settlement process, we conducted an in-depth study of an illustrative case. A recent OIG settlement related to an EMTALA violation involving an OB emergency was identified. Reports and proceedings from the EMTALA investigation including the facility’s proposed corrective actions were obtained from CMS via a Freedom of Information Act request. Individual patient-level identifiers were redacted in documents provided. We examined contextual information about the hospital cited for this EMTALA violation to provide understanding of the circumstances and conditions in which the hospital operates. The clinical case that led to the EMTALA investigation was described in detail. We summarized EMTALA investigation findings and facility corrective actions from this case to provide a deeper example of the EMTALA enforcement process and hospital response to EMTALA citation for cases involving labor and OB emergencies.

## RESULTS

### Characteristics of Civil Monetary Penalties Related to Obstetrical Emergencies

**Figure 1:**
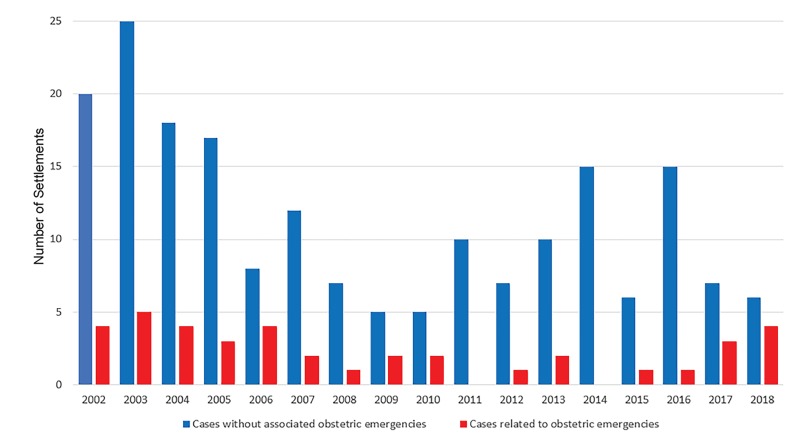
Civil monetary penalty settlements related to violation of the Emergency Medical Treatment and Labor Act Involving obstetrical emergencies by year (number).

Between 2002–2018, there were 232 civil monetary penalty settlements related to EMTALA in the US. Among these, eight (3%) were levied against individual physicians and 224 (97%) were levied against facilities. Of all civil monetary penalty settlements related to EMTALA, 39 (17%) involved OB emergencies, including three against individual physicians. The kappa inter-rater reliability for identification of OB cases was 0.985. (The sole case with disagreement upon preliminary review was determined by consensus to be related to an OB condition). While the number of overall annual EMTALA-related settlements declined by 58% during the study period from 24 in 2002 to 10 in 2018, settlements related to labor and other OB emergencies occurred relatively consistently (Figure 1), with four settlements in the first and last years of the study period. The proportion of all settlements related to labor and other OB emergencies increased from 17% in the first year to 40% in the final year of the study period.

Most cases resulting in settlements involving OB emergencies centered on a failure to provide medical screening exam (82%) and/or stabilizing treatment (51%). Failure to arrange appropriate transfer was more common for OB-related settlements (36%) compared with non-OB settlements (21%) (p = 0.041). Failure to accept an appropriate transfer (5%) and failure of an on-call doctor to respond (3%) were less common in OB cases. Characteristics of OIG settlements related to EMTALA violations involving OB emergencies are shown in Table 1.

Although location of incident was not uniformly recorded, 21 (54%) cases were specifically noted to have occurred in an ED compared with five (13%) in labor and delivery areas. Additionally, six (15%) of the settlements involving OB issues included descriptions of EMTALA deficiencies related to separate patients with non-OB complaints. (See Appendix C for example). Fifteen (38%) OB settlements were noted to involve a provider specifically directing a pregnant woman to proceed to another hospital, with seven (47%) of these women directed specifically to hospitals where their obstetrician practiced. Nine (60%) of these patients were specifically noted to proceed to the other hospital by private vehicles. In one case a patient was escorted to their personal vehicle and directed to call 911. While ages of patients involved in cases resulting in civil monetary penalties are not systematically reported, seven (18%) settlements related to OB emergencies were specifically noted to involve a pregnant minor. Settlement summaries for those cases noted to involve a pregnant minor are included in Appendix C.

**Figure 2:**
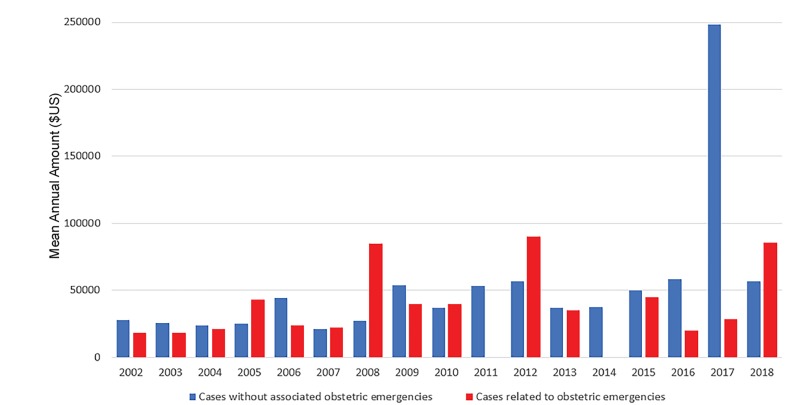
Civil monetary penalty settlements related to violations of the Emergency Medical Treatment and Labor Act Involving Obstetrical Emergencies, mean annual amount ($US).

Of the 39 civil monetary penalties related to OB emergencies, 15 (38%) occurred in CMS Region IV, including eight (53%) in Florida and five (20%) in North Carolina. CMS Region VI accounted for eight (21%) settlements related to OB emergencies with five (63%) of these in Texas, and three (37%) in Louisiana. Average settlements related to OB emergencies by year are depicted in Figure 2. For the majority of the study period, the maximum OIG civil monetary penalty for an EMTALA violation was set at $50,000, which approximately doubled in 2016 with plans for future inflation adjustments.^20^ Four settlements exceed the maximum penalty amount, including for $80,000 in 2005, $85,000 in 2008, $90,000 in 2012, and $200,000 in 2018, indicating that the OIG has been stacking penalties for multiple deficiencies identified during a single citation event.

### Case Study

**Figure 3:**
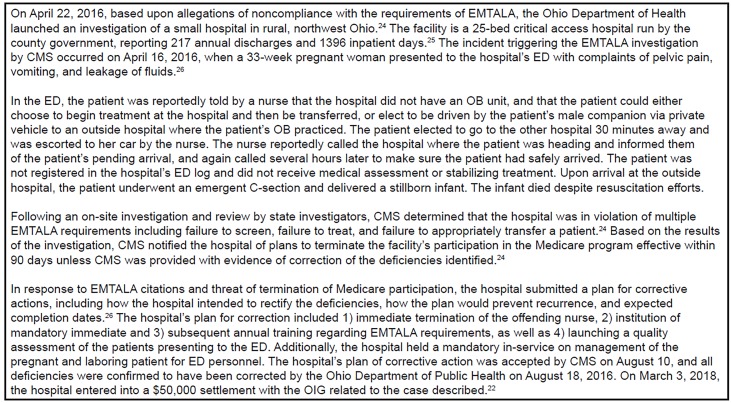
Illustrative case study. *EMTALA*, Emergency Medical Treatment and Labor Act; *OIG*, Office of the Inspector General; *CMS*, Centers for Medicare and Medicaid Services.

To provide a richer description of the EMTALA enforcement process and hospital response to EMTALA citations, we included findings and facility corrective actions from the EMTALA investigation related to a recent OIG settlement involving an OB emergency in Figure 3.

## DISCUSSION

Maternal mortality rates in the US are rising, and now exceed those of other developed countries,^21^ indicating significant room for improvement in OB care. More than three decades after EMTALA was passed and despite inclusion of the term “labor” in the law’s title, hospitals continue to be cited and fined for EMTALA cases related to labor and other OB emergencies. Since 2002, the OIG has reached 39 civil monetary penalty settlements related to EMTALA violations involving labor and other OB emergencies, including three against individual physicians. While the number of annual settlements for EMTALA cases declined by more than 50% over the study period, cases related to OB emergencies remained consistent.

The proportion of settlements involving OB emergencies increased from 17% to 40% between 2002–2018. Generally, civil monetary penalties for EMTALA violations related to OB emergencies tended to involve failure to provide medical screening exam and stabilization and to concentrate in a few CMS regions. OB settlements were significantly more likely than non-OB settlements to involve failure to arrange an appropriate transfer. Nearly one in five OB cases involved a pregnant minor. Study findings highlight a number of key points important for hospital administrators, emergency physicians, and OB providers to be aware of.

Among civil monetary penalty settlements involving labor and OB emergencies, failure to provide appropriate screening exam was the most commonly cited cause for EMTALA citation, identified in 87% of cases. Under EMTALA, any patient presenting to the ED must be screened for evidence of an emergent condition or active labor. According to CMS, labor is defined to mean the process of childbirth beginning with the latent or early phase of labor and continuing through delivery of the placenta.^27^ CMS further clarifies that a woman experiencing contractions is considered to be in true labor, unless after a reasonable observation period, a qualified medical provider certifies that the woman is in false labor.^27^ The medical provider (a physician, certified nurse-midwife, or other qualified medical personnel acting within his or her scope of practice as defined in hospital staff bylaws and state law) must also complete a reasonable observation period.

We found that 13% of settlements were specifically noted to involve labor and delivery triage areas. While it is commonly understood that EMTALA applies to patients presenting to medical EDs, it is important for providers to understand that many labor and delivery evaluation areas that evaluate patients for emergent conditions on an unscheduled basis qualify as dedicated EDs and are required to comply with screening, stabilization, and transfer requirements of EMTALA, if located within a hospital with a Medicare provider agreement.^28^

The importance of providing appropriate care to pregnant minors should be highlighted. Nearly one in five of the OB settlements involved a pregnant minor, and 86% of these cases centered failure to provide appropriate medical screening exam for the pregnant minor (Appendix C). CMS has clarified that under EMTALA, a minor can request an examination or treatment for an emergency medical condition, and that a hospital is required by law to conduct the exam to determine whether an emergency medical condition exists.^27^ Medical screening exams or treatment of an emergent condition should not be delayed by waiting for parental consent.

Failure to provide appropriate stabilizing treatment was the second most commonly cited cause for EMTALA citation leading to OIG settlements among patients with OB emergencies, identified in more than half of cases. An individual is considered stabilized if the treating provider has determined with reasonable clinical confidence, that the emergency medical condition has been resolved.^27^ In the case of active labor, medically stabilization is achieved when a woman has delivered the child and the placenta.^27^ According to CMS for patients requiring transfer, stabilized is defined as “no material deterioration of the condition is likely, within reasonable medical probability, to result from or occur during the transfer of the individual from a facility.” EDs at hospitals without dedicated OB services must still provide stabilizing treatments to laboring women under EMTALA and should implement policies for evaluation and stabilization of pregnant patients.

More than a third of OIG settlements in this study were cited for failure to arrange appropriate transfer compared with only a fifth of non-OB settlements. According to CMS, if a woman is in labor the hospital must deliver the baby and the placenta or transfer appropriately.^27^ Study findings and the illustrative case highlight the need for EDs to follow EMTALA requirements for appropriate transfer of patients in active labor even if dedicated OB services are unavailable at the hospital. This is particularly important as 45% of rural counties in the US had no OB services between 2004–2014, and an additional 9% of rural counties lost OB services during that period, leaving more than half of US rural counties without hospital OB services.^29^ In the illustrative case described, an ED nurse informed the patient that OB services were not available at the hospital and offered for the pregnant woman to proceed via private vehicle to the facility where her obstetrician practiced, even calling to inform the intended receiving hospital to expect the patient.

The offer, suggestion, or demand by hospital staff for pregnant patients to proceed via private vehicle to another facility, typically the hospital where their obstetrician practiced, was a common theme noted among settlements involving OB emergencies. EMTALA requires any patient presenting to a dedicated ED to be entered into a log, have a documented screening exam, stabilization, and when indicated appropriate transfer for specialty care even if the most logical and reasonable course of action might seem to be for a patient to be transported via private vehicle to a facility that has the specialty services that they require. The transferring hospital must provide treatment within its on-site capability that minimizes the risks of the woman and the unborn child, obtain permission from the receiving hospital for transfer, and send medical records with the patient.^27^ The sending hospital is responsible for ensuring that the transfer is effected through qualified personnel and transportation equipment including the use of medically appropriate life support measures during transfer.^27^

Additionally, CMS has specified that a pregnant patient in labor may not be transferred unless she, or a legally responsible person acting on her behalf, requests a transfer and a physician or other qualified medical personnel, in consultation with a physician, certifies that the benefits to the woman and/or the unborn child outweigh the risks associated with transfer.^27^ Had the provider in the illustrative case logged the visit and provided a medical screening exam, they would have had sufficient information to either provide stabilizing services and arrange appropriate transfer, or to adequately and appropriately inform the patient of the risks and benefits of leaving the hospital if the patient were to decline stabilizing services at the original facility.

Failure to accept an appropriate transfer (5%) and failure of an on-call doctor to respond (3%) were relatively rare in the current study. While hospitals with on-call obstetricians without a dedicated ED may not be obligated to adhere to certain aspects of EMTALA (eg, providing medical screening exams, stabilizing treatment), it is worth noting that they are required to accept appropriate transfer of patients from another dedicated ED with emergent OB conditions requiring specialized treatment if the hospital has a Medicare provider agreement.

OIG settlements related to OB conditions concentrate in two of the 10 CMS regions (IV and VI), with a third of cases occurring in Florida and Texas. This is consistent with prior published work showing both high rates of EMTALA-related OIG settlements in the same regions.^6^ Both Florida and Texas have maternal mortality ratios far above the national average^30^ suggesting that the quality of OB care may be contributory. Further work is needed to determine whether the high rates of civil monetary penalty settlements reflect suboptimal OB emergency care or enhanced enforcement in CMS regions IV and VI.

## LIMITATIONS

Although this study provides the most comprehensive assessment to date of OIG penalties resulting from EMTALA citations related to OB emergencies, there are a number of potential limitations. First, as reported findings rely upon administrative data provided by the OIG, data may be limited by variability in reporting and enforcement of EMTALA cases related to OB emergencies across regions or over time. However, the case descriptions analyzed represent the best available data to study OIG penalties. While it would be ideal to report overall trends in EMTALA enforcement for OB emergencies, available data for EMTALA citations not resulting in fines reported by CMS does not provide granular details about cases included in settlement descriptions.

While additional documentation related to EMTALA settlements involving OB emergencies were requested via the Freedom of Information Act for a more in-depth qualitative review, only a limited number of documents were available at the time of submission and were included in the illustrative case study. While it would have been ideal to separately analyze settlements related to labor and other OB emergencies, many of the case descriptions were sufficiently vague such that it was impossible to determine whether a pregnant patient was in labor or not at the time of the alleged incident; thus, all OB cases were grouped. Second, available data is limited to EMTALA cases resulting in civil monetary penalty settlement agreements.

Finally, published settlement descriptions varied markedly in detail and some descriptions were sufficiently vague such that settlements related to OB emergencies may not have been identified (eg, “The OIG alleged that the hospital failed to provide appropriate medical screening examinations and stabilizing treatment to two patients.”) However, in the vast majority of OIG settlement descriptions, the nature of the condition was indicated, and the proportion of settlements related to OB emergencies (17%) was similar to the proportion of overall EMTALA citations involving labor and OB emergencies identified previously (14%).^6^

## CONCLUSION

Despite inclusion of the term “labor” in the title of the law, approximately one in six civil monetary penalty settlements related to EMTALA violations involve OB emergencies. While the overall number of annual settlements declined during the study period, settlements related to OB emergencies occurred consistently throughout, accounting for 17% of settlements in 2002 and 40% in 2018. Our study found that failure to arrange appropriate transfer was more common among OB settlements and that settlements related to OB conditions concentrate in two of the 10 CMS regions. One in five cases was specifically noted to involve a pregnant minor, indicating that emergency physicians and obstetricians may benefit from education regarding obligations to evaluate, stabilize, and when necessary arrange for appropriate transfer of pregnant minors with active labor or other OB emergencies, even absent parental consent. Recent cases highlight the need for hospital administrators, emergency physicians, and obstetricians to evaluate and strengthen policies and procedures related to both screening exams and stabilizing care of patients with labor and OB emergencies, even if the hospital does not provide dedicated OB care.

## Supplementary Information







## Figures and Tables

**Table 1:** Characteristics of EMTALA-related civil monetary penalty settlements involving obstetrical emergencies.

	Obstetrical		Non-obstetrical		P-value	Test type
Total number	39		193			
Settlement (mean US dollars)	$36,269.23		$43,677.87		0.6386	Student’s t-test
	n	%	n	%		
Settlement against physican	3	8%	5	3%	0.134	Fischer's exact test
Minor involved	7	18%	24	12%	0.356	Pearson Chi squared
Failure to MSE	32	82%	142	74%	0.265	Pearson Chi squared
Failure to stabilize	20	51%	105	54%	0.721	Pearson Chi squared
Failure to arrange transfer	14	36%	40	21%	0.041	Pearson Chi squared
Failure to accept transfer	2	5%	29	15%	0.123	Fischer's exact test
On call failed to respond	1	3%	13	7%	0.475	Fischer's exact test
CMS region					0.052	Fischer’s exact test
1	2	5%	5	3%		
2	0	0%	8	4%		
3	3	8%	1	1%		
4	15	38%	81	42%		
5	4	10%	20	10%		
6	8	21%	20	10%		
7	2	5%	25	13%		
8	0	0%	6	3%		
9	5	13%	27	14%		
10	0	0%	0	0%		

*EMTALA*, Emergency Medical Treatment and Labor Act; *OIG*, Office of the Inspector General; *MSE*, medical screening exam; *CMS*, Centers for Medicare and Medicaid Services.
