# Supplementary material for: Penalties for Emergency Medical Treatment and Labor Act Violations Involving Obstetrical Emergencies
Source: West J Emerg Med. 2020 Feb 21;21(2):235–43. doi: 10.5811/westjem.2019.10.40892 (PMC7081879; doi:10.5811/westjem.2019.10.40892)
Supplement: Supplementary file 1 [file wjem-21-235-s001.docx]

**Appendix A:** Centers for Medicare & Medicaid Services (CMS) Regional Map


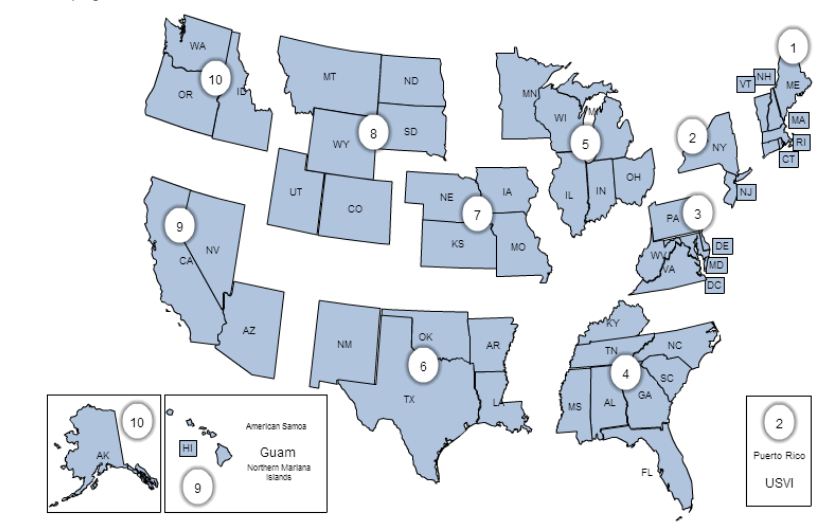


Source: Centers for Medicare and Medicaid Services. Available at: <https://www.cms.gov/About-CMS/Agency-Information/RegionalOffices/RegionalMap.html>) Accessed December 11, 2018.
