# Supplementary material for: Penalties for Emergency Medical Treatment and Labor Act Violations Involving Obstetrical Emergencies
Source: West J Emerg Med. 2020 Feb 21;21(2):235–43. doi: 10.5811/westjem.2019.10.40892 (PMC7081879; doi:10.5811/westjem.2019.10.40892)
Supplement: Supplementary file 2 [file wjem-21-235-s002.docx]

**Appendix B:** EMTALA Clinical Deficiency Tags and Summary of EMTALA Interpretive Guidelines^1^

| **Deficiency Tag** | **Guideline Code** | **Description** |
| --- | --- | --- |
| 2404 | §489.24(j) | Availability of On-Call Physicians |
| 2406 | §489.24(a); §489.24(c) | Appropriate Medical Screening Examination |
| 2407 | §489.24(d)(1,2,3) | Stabilizing Treatment |
| 2409 | §489.24 (e)(1,2) | Appropriate Transfer |
| 2411 | §489.24(f) | Recipient Hospital Responsibilities |
| EMTALA violations involving clinical cases are forwarded to the OIG for review and consideration for penalties; therefore this list contains EMTALA deficiency tags pertaining to clinical care. This list excludes EMTALA deficiency tags related to administrative components of the law (e.g. sign posting). | | |

Source:

1. CMS Manual System Pub. 100-07 State Operations Provider Certification: Revisions to Appendix V, "Emergency Medical Treatment and Labor Act (EMTALA) Interpretive Guidelines". In: DHHS, ed.2009.
