# Supplementary material for: Penalties for Emergency Medical Treatment and Labor Act Violations Involving Obstetrical Emergencies
Source: West J Emerg Med. 2020 Feb 21;21(2):235–43. doi: 10.5811/westjem.2019.10.40892 (PMC7081879; doi:10.5811/westjem.2019.10.40892)
Supplement: Supplementary file 3 [file wjem-21-235-s003.docx]

**Appendix C:** Civil Monetary Penalties Related to EMTALA Violations Involving Pregnant Minors, 2002-2018

| All obstetrical-related settlement entries from the OIG involving pregnant minors is provided. Specific identifiers have been removed, but are available online.^1-3^ | |
| --- | --- |
| Date | Settlement Description |
| 03-23-2018 | [Hospital name redacted], Port Charlotte, Florida, entered into a $42,500 settlement agreement with OIG. The settlement agreement resolves allegations that based on OIG's investigation, [hospital name redacted] violated EMTALA when it failed to accept an appropriate transfer of a patient. Specifically, the patient, a 17-year-old female, presented to another hospital's ED, 6 weeks pregnant, complaining of abdominal pain continuing for approximately one week. An ultrasound confirmed the intrauterine fetus with a heartbeat and a left ectopic ruptured mass. The patient needed obstetric care, which was not available at that hospital. Accordingly, the ED contacted the Hospital Corporation of America Transport Center (TC) and requested transfer for a possible ruptured ectopic pregnancy. When TC communicated to [hospital name redacted]’s ED that it was trying to facilitate an ED-to-ED transfer, [hospital name redacted]’s representative replied that it did not accept ED-to-ED transfers and hung up. TC called [hospital name redacted] back and the call was transferred to the ED, where it was reiterated that [hospital name redacted] did not accept ED-to-ED transfers. The ED employee also mentioned that they had given the transferring hospital the contact information for [hospital name redacted]’s on-call OB/GYN. The transferring hospital called [hospital name redacted]’s on-call OB/GYN who requested all of the patient medical reports be faxed to him before he would consider accepting the patient. During this conversation the doctor learned that the patient was "out of county" and he did not accept the transfer. |
| 12-04-2017 | [Hospital name redacted], Greenville, Alabama, entered into a $20,000 settlement agreement with OIG. The settlement agreement resolves allegations that, based on OIG's investigation, [hospital name redacted] violated EMTALA when it failed to provide an adequate MSE and stabilizing treatment for a 16-year-old female patient. The patient was 27-weeks pregnant and had presented by ambulance to [hospital name redacted]’s ED, complaining of vaginal bleeding and a sharp pain in her lower abdomen. A nurse obtained the patient's vital signs and measured the fetal heart rate. An ED physician examined the patient and called the patient's obstetrician to discuss the patient's condition. Without providing an appropriate MSE, the ED physician decided to send the patient for monitoring to another hospital where the patient's obstetrician was located. The ED physician discharged the patient and instructed her to go to the other hospital that was 55 miles away from [hospital name redacted]. On the way to the other hospital, the patient's family members called EMS. When EMS arrived, the patient was lying on the ground next to her car, experiencing severe abdominal pain, vaginal pain, and light bleeding. EMS drove the patient to a different hospital where she delivered a stillborn infant within minutes of arriving. |
| 06-12-2006 | [Hospital name redacted], Florida, agreed to pay $20,000 to resolve its liability for CMPs under the patient dumping statute. The OIG alleged that [hospital name redacted] failed to provide appropriate MSEs and/or stabilizing treatment for two patients who went to [hospital name redacted]’s ED. The mother was allegedly informed that [hospital name redacted] did not treat pediatric patients and that she would have to take her daughter to another facility. The daughter, who was pregnant, presented to another facility with lower abdominal pain and vaginal bleeding. She was stabilized and transported to another hospital. The second patient presented to [hospital name redacted]’s ED accompanied by fire rescue workers, the police, and his grandmother. The patient was threatening to burn himself. A psychiatric nurse allegedly suggested that the patient be taken to another hospital across the street, where beds were readily available, in order to avoid a long wait in the ED. The patient was taken to the other hospital where he was admitted. |
| 03-15-2006 | [Hospital name redacted], Florida, agreed to pay $15,000 to resolve its liability for CMPs under the patient dumping statute. The OIG alleged that the hospital failed to provide an appropriate MSE to a pregnant minor who presented to its ED with complaints of stomach pain and pressure, and blood in her urine. The hospital refused to treat the patient without parental consent. |
| 08-30-2004 | [Name redacted], a small Louisiana hospital, agreed to pay $15,000 to resolve its liability for CMPs under the patient dumping statute. The OIG alleged that the hospital failed to provide an appropriate MSE and stabilizing treatment to a pregnant 17-year-old female who presented to the hospital's ED with complaints of perineal numbness and vaginal bleeding. A physician refused to treat her due to his erroneous belief that he could not do so absent parental consent. |
| 06-23-2004 | A Louisiana physician agreed to pay $10,000 to resolve his liability for CMPs under the patient dumping statute. The OIG alleged that the physician failed to provide an appropriate MSE and stabilizing treatment to a pregnant 17-year-old female who presented to the hospital's ED with complaints of perineal numbness and vaginal bleeding. The physician refused to treat her due to his erroneous belief that he could not do so absent parental consent. |
| 03-13-2003 | [Name redacted] a small Pennsylvania hospital, agreed to pay $5,000 to resolve its liability for CMPs under the patient dumping statute. The OIG alleged that [name redacted] failed to provide a 17-year-old pregnant female in labor an appropriate MSE before instructing her to proceed to another hospital 15 miles away, where her doctor had admitting privileges. The patient arrived at the other hospital prior to delivery and the child was safely delivered. |
| Key | ED: Emergency department, CMP (civil monetary penalty), MSE Medical screening exam ; EMS emergency medical services |

Source:

1. Office_of_Inspector_General_U.S._Department_of_Health_&_Human_Services. Civil Monetary Penalties and Affirmative Exclusions. Available at: <https://oig.hhs.gov/fraud/enforcement/cmp/background.asp>. Accessed September 1, 2018.

2. Office_of_Inspector_General_U.S._Department_of_Health_&_Human_Services. Civil Monetary Penalties: Patient Dumping. Available at: <http://oig.hhs.gov/fraud/enforcement/cmp/patient_dumping.asp>. Accessed December 9, 2015.

3. Office_of_Inspector_General_U.S._Department_of_Health_&_Human_Services. Patient Dumping Archives. Available at: <http://oig.hhs.gov/reports-and-publications/archives/enforcement/patient_dumping_archive.asp>. Accessed March 7, 2016.
